# Translocation of the retinal pigment epithelium and formation of sub-retinal pigment epithelium deposit induced by subretinal deposit

**Published:** 2007-06-14

**Authors:** Lian Zhao, Zhenfang Wang, Yun Liu, Ying Song, Yiwen Li, Alan M. Laties, Rong Wen

**Affiliations:** 1 Department of Ophthalmology, University of Pennsylvania, School of Medicine, Philadelphia, PA; 2 Department of Eye Trauma, Zhongshan Ophthalmic Center, Guangzhou, Guangdong 510060, China

## Abstract

**Purpose:**

A cardinal pathological feature of age-related macular degeneration (AMD) is the deposition of extracellular material between the retinal pigment epithelium (RPE) and Bruch's membrane, pathologically described as sub-RPE deposits. Both the presence and local organization of these deposits contribute to the clinical manifestations of AMD, including localized deposits clinically recognized as drusen. The biogenesis of sub-RPE deposits remains elusive. This work explores the pathological processes of sub-RPE deposit formation.

**Methods:**

Matrigel was injected to the subretinal space of rats to create an amorphous deposit. Tissue sections were examined by light or confocal microscopy.

**Results:**

In the presence of the subretinal deposit of Matrigel, RPE cells leave Bruch's membrane to migrate toward photoreceptors and then form a new layer between the deposit and photoreceptors, resulting in RPE translocation. The new RPE layer displaces the deposit to the sub-RPE location and therefore it becomes a sub-RPE deposit. The RPE mobilization requires the presence of photoreceptors. Bruch's membrane devoid of RPE attachment becomes vulnerable to invasion by new blood vessels from the choroid.

**Conclusions:**

Our work supports a novel model of sub-RPE deposit formation in which excessive material first accumulates in the subretinal space, disrupting the physical contact between RPE cells and photoreceptors. To restore the contact, RPE cells migrate toward photoreceptors and form a new layer. The subretinal material is consequently displaced to the sub-RPE location and becomes sub-RPE deposit. Our data also provide evidence that the presence of sub-RPE deposit is sufficient to induce choroidal neovascularization to penetrate Bruch's membrane.

## Introduction

Age-related macular degeneration, a leading cause of irreversible blindness in people over age 65 years [1-3], is characterized by soft drusen, retinal pigmentary disturbances, and/or focal retinal atrophy. Pathologically, drusen are localized sub-retinal pigment epithelium (sub-RPE) deposits, abnormal accumulation of extracellular material located between the RPE and Bruch's membrane [2]. Considerable information has been accumulated on the morphology, ultrastructure, and molecular constituents of these deposits. However, the source(s) of sub-RPE deposits and how they are formed remain enigmatic [2,4-7]. RPE cells were suspected to give rise to drusen when they were first described some 150 years ago [8,9]. Later, choroid was suggested as their source [10]. Local immune-mediated processes were implicated in drusen biogenesis as well [5]. Animals that lack monocyte chemoattractant protein-1 (MCP-1, also known as CCP-1) or its cognate C-C receptor-2 (Ccr-2) [11], or that lack collagen XVIII/endostatin [12] are shown to develop sub-RPE deposits/drusen. Recent findings that variants in complement factor H [13-16], B, complement component 2 (C2) [17], and LOC387715/HTRA1 [18,19] genes may increase the risk to develop age-related macular degeneration (AMD) provide insight into the pathological process of AMD.

The exudative form of AMD, a major cause of severe vision loss, is characterized by choroidal neovascularization (CNV) as new blood vessels from the choroid penetrate Bruch's membrane to enter the retina. The mechanism of CNV development is not fully understood. Pathological studies have demonstrated a strong association between sub-RPE deposits and CNV [20-23]. In addition, focal inflammation has been found to induce focal thinning and breaks of Bruch's membrane in patients with AMD, along with a phenotypic switch of local choroidal endothelial cells from quiescent to angiogenic [24,25], suggesting the involvement of sub-RPE deposits. Nevertheless, it is not clear whether sub-RPE deposits and CNV are simply two independent manifestations of the same disease, or if the deposits play a role in CNV development.

Using Matrigel to create an amorphous deposit in the subretinal space in rats, we demonstrate that RPE translocation displaces the Matrigel deposit to the sub-RPE location. Subsequently, new blood vessels from the choriocapillaris penetrate Bruch's membrane and enter the Matrigel deposit. These data support a novel mechanism of sub-RPE deposit formation in which RPE translocation plays a central role in converting subretinal material into sub-RPE deposits.

## Methods

### Animals and subretinal injections

All procedures involving animals were approved by the Institutional Animal Care and Use Committee of the University of Pennsylvania and adhered to the Association for Research in Vision and Ophthalmology Statement for the Use of Animals in Ophthalmic and Vision Research. Adult Sprague-Dawley and Long Evans rats (female, 2-3 months old) were purchased from Harlan Laboratories (Indianapolis, IN). Pigmented heterozygous transgenic rats carrying a murine rhodopsin mutation S334ter were produced by mating homozygous breeders of male S334ter transgenic rats (Sprague-Dawley background) with wild-type Long Evans females.

**Figure 1:**
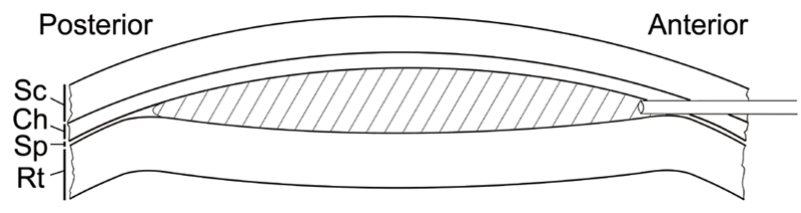
Schematic illustration of subretinal injection. The tip of a blunt needle is introduced to the subretinal space (Sp) at a shallow angle toward the posterior pole. Injected material formed a bleb (shade area) in the subretinal Sp. Sc indicates sclera; Ch indicates choroid; Rt indicates retina.

Subretinal injections of Matrigel (growth factor reduced, BD Biosciences, Bedford, MA) were performed on the temporal side of an eye under a microscope. A rat was anesthetized by intraperitoneal injection of ketamine (40 mg/kg) and xylazine (6 mg/kg). The sclera was exposed and an incision was made between the limbus and the equator with a sharp number 33 needle to reach the subretinal space. The tip of a blunt number 33 needle attaching to a Hamilton micro-syringe was introduced to the incision at a 5 degree angle toward the posterior pole and inserted 0.1-0.2 mm into the subretinal space. Matrigel was diluted with phosphate buffered saline (PBS) at 3:1 (75% gel). Gel solution (1.2 μl) was slowly injected so the solution pushed its way into the subretinal space. Injected solution normally solidified within minutes and formed a bleb of 1.5-2 mm in diameter with the injection site at the anterior edge (Figure 1).

### Histology

Animals were sacrificed by CO_2_ overdose and perfused with 2% paraformaldehyde and 2.5% glutaraldehyde (in 0.1 M phosphate buffer, pH 7.4). Eyes were collected and post-fixed in the same fixative. The posterior segments of the eyes were embedded in an Epon-Araldite mixture [26]. Semi-thin sections (1 μm) were cut through Matrigel injected areas, stained with toluidine blue, and examined by light microscopy.

### Visualization of blood vessels

Blood vessels were directly labeled with a solution containing a fluorescent carbocyanine dye DiI (1, 1'-Dioctadecyi-3, 3, 3', 3'-tetramethylindocarbocyanine perchlorate, Sigma-Aldrich, St. Louis, MO). An animal was sacrificed by CO_2_ overdose and perfused with PBS (4-5 ml), followed by a solution containing 160 mM DiI (4-5 ml). The animal was subsequently perfused with 4% paraformaldehyde (20 ml in 0.1M phosphate buffer, pH 7.4). Eyes were harvested and the anterior segments removed. The eyecups were post-fixed in the same fixative overnight and then transferred to PBS at 4 °C. Tissue was embedded in 5% agarose. Thick (100 μm) serial sections were cut on a vibratome (VT1000S, Leica Microsystems, Bannockburn, IL), mounted on glass slides with 80% glycerol, and examined by confocal microscopy.

## Results

### Retinal pigment epithelium cell migration after subretinal injection of Matrigel

**Figure 2:**
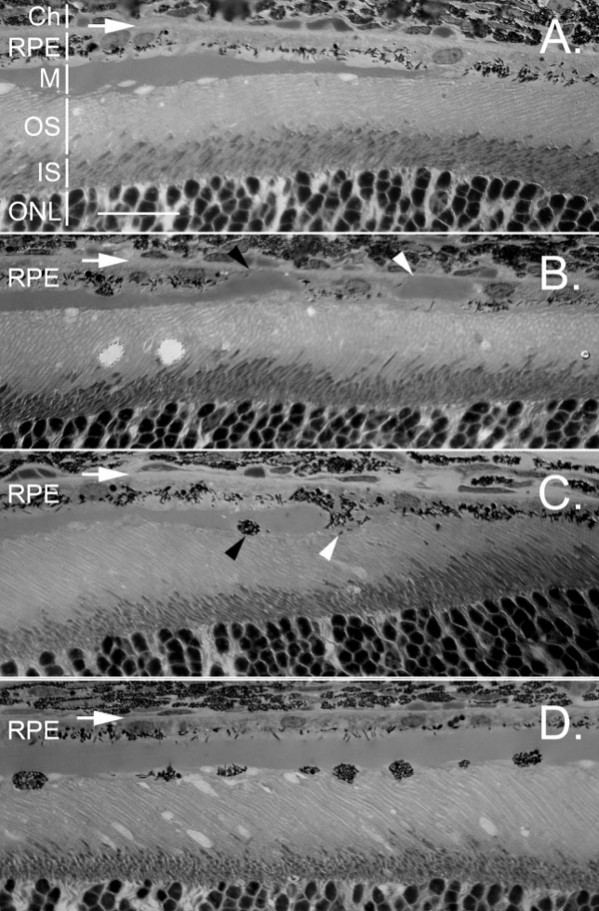
Retinal pigment epithelium cells mobilization in response to subretinal Matrigel. The position of Bruch's membrane is indicated by a horizontal white arrow in each panel. **A**: The edge of Matrigel layer in the subretinal space is shown 1 day after injection. No retinal pigment epithelium (RPE) cells mobilization is detected. RPE mobilization was detected 5 days after injection of Matrigel (**B**-**D**). In **B**, a small piece of gel (white arrowhead) had been relocated to the sub-RPE space. On the left side of the same retinal section, two RPE cells formed a gap, entrapping the tip of the main gel body (black arrowhead). The gel tip was thus in direct contact with Bruch's membrane. **C**: A cell at the edge of the gel (white arrowhead) extended between photoreceptors and the gel. Another had migrated to the photoreceptor side of the gel (black arrowhead). **D**: In the central region of the Matrigel layer, many cells had migrated to the photoreceptor side. Ch indicates choroid; M indicates Matrigel layer; IS indicates inner segments; ONL indicates outer nuclear layer. The scale bar represents 20 μm.

After injection, Matrigel formed a layer between photoreceptors and the RPE (Figure 2A). RPE cells remained in their original position attached to Bruch's membrane. Some small vesicles appeared between the tips of photoreceptor outer segments and the inner surface of the Matrigel layer (Figure 2A).

Active mobilization of RPE cells was seen as early as 5 days after injection of Matrigel (Figure 2B-D). In Figure 2B, a small piece of gel (Figure 2B, white arrowhead) had been relocated to the space between the RPE and Bruch's membrane, covered by two RPE cells. This piece of gel thus became a sub-RPE deposit. The relocation process is vividly displayed in the same retinal section. Two RPE cells close to the tip of the of the main gel body (Figure 2B, black arrowhead) had dissociated from each other and partially detached from Bruch's membrane to form a gap. The tip of the gel was entrapped in the gap and was in direct contact with Bruch's membrane. The cell at the gel tip was extending into the space between photoreceptors and the gel (Figure 2B).

At the edge of the Matrigel deposit, RPE cells circumvented the gel by extending into the space between photoreceptors and the gel (white arrowhead, Figure 2C), or directly migrating to the photoreceptor side of the gel (black arrowhead, Figure 2C). In the central region of the gel body, RPE cells directly migrate to the photoreceptor side (Figure 2D).

### Formation of a new retinal pigment epithelium layer after injection of Matrigel

**Figure 3:**
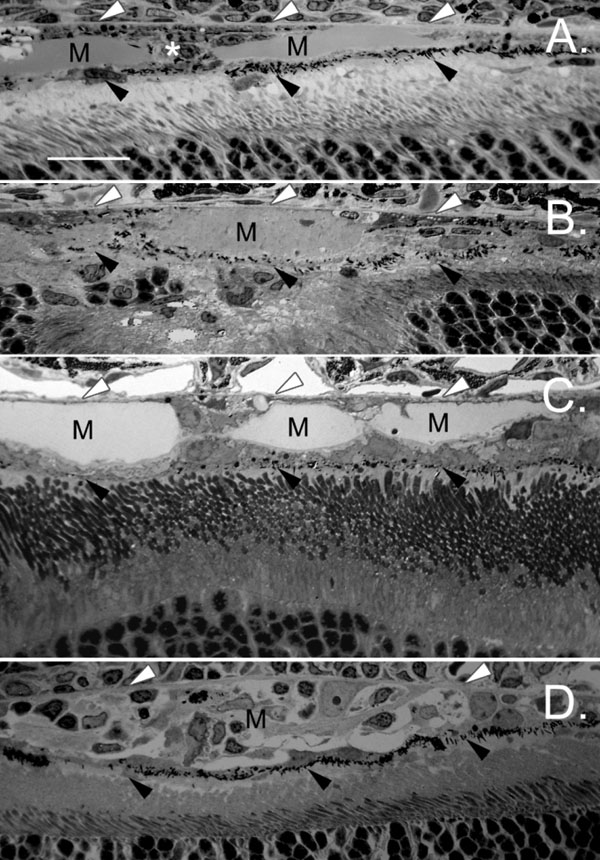
Formation of a new retinal pigment epithelium layer. **A**: A new monolayer (black arrowheads) formed between the Matrigel (M) and photoreceptors 10 days after Matrigel injection, displacing the Matrigel to the sub-RPE location. Comparable new retinal pigment epithelium (RPE) layers (black arrowheads) were seen (Bruch's membrane is indicated by white arrowheads) 25 days (**B**), 30 days (**C**), and 45 days (**D**) after Matrigel injection. In some cases, the Matrigel injected area (M) was filled with cells to form scare-like tissue (**D**). The scale bar represents 20 μm.

After migrating to the photoreceptor side, RPE cells formed a monolayer and reestablished direct contact with photoreceptors. The formation of a new RPE layer occurred as early as 10 days after Matrigel injection (Figure 3A). Some cells formed a bridge between Bruch's membrane and the new RPE layer, perhaps in the process of migrating. A few cells remained in the original location, attached to Bruch's membrane, (Figure 3A). Comparable new RPE layers were found 25 days (Figure 3B), 30 days (Figure 3C), and 45 days (Figure 3D) after Matrigel injection, indicating that the migration stabilized once the new layer had been formed. In some cases, the Matrigel deposit was infiltrated by cells to form a scar-like structure (Figure 3D). Shortening of photoreceptor outer segments and certain degree of photoreceptor degeneration were also seen (Figure 3).

### Photoreceptors are necessary for retinal pigment epithelium migration

**Figure 4:**
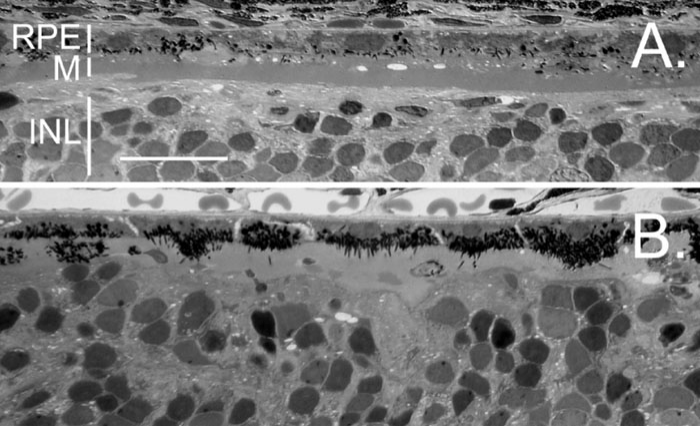
No retinal pigment epithelium translocation in the absence of photoreceptors. Eyes were obtained from the S334ter transgenic rats 15 days (**A**) or 45 days (**B**) after Matrigel injection. No photoreceptors or outer nuclear layer were identifiable. The Matrigel layer (M) was between the retinal pigment epithelium (RPE) and the rest of the neuronal retina. RPE cells attached to Bruch's membrane. No obvious RPE migration is detected. No new RPE layer formed on the retinal side of the Matrigel layer. Significant hyperpigmentation occurred in the RPE (**B**). INL indicates inner nuclear layer. The scale bar represents 20 μm.

The above findings indicate that when Matrigel disrupts the direct RPE-photoreceptor contact, RPE cells circumvent the Matrigel layer to reestablish the contact. We hypothesize that the directional migration of RPE cells is chemotactic in nature and photoreceptors are the target. To test this hypothesis, we used animals with virtually no photoreceptors. Transgenic rats carrying the rhodopsin mutation S334ter undergo rapid photoreceptor degeneration: more than 90% of photoreceptors are lost by postnatal day (PD) 20 and almost all are degenerated by PD30 [27,28]. We therefore chose PD30 animals for subretinal injection of Matrigel. RPE cells in these animals remained in their original location 15 days after Matrigel injection (Figure 4A). No RPE migration occurred even 45 days after injection (Figure 4B). Thus, RPE migration and translocation require a signal from photoreceptors. We cannot rule out the possibility that RPE cells might have changed in these animals in the absence of photoreceptors.

### Induction of choroidal neovascularization after injection of Matrigel

We used this model to investigate the role of sub-RPE deposits in CNV development. To avoid any new blood vessels that might have been induced by the needle injury to Bruch's membrane during injection, we looked for new blood vessels traversing Bruch's membrane in a region distinct from the injection site. Since the posterior half of the injected area was unambiguously separate from the injection site and not touched by the injecting needle (Figure 1), we were specifically looking for new blood vessels penetrating Bruch's membrane in the posterior half of the Matrigel area.

**Figure 5:**
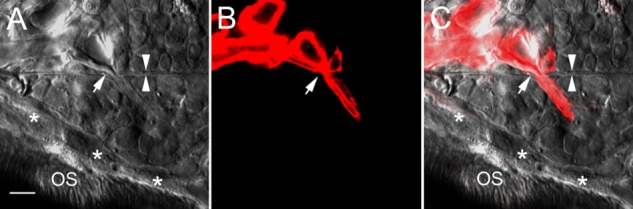
Choroidal neovascularization induced by Matrigel deposit. **A**: A differential interference contrast (DIC) image shows the posterior edge of a Matrigel deposit. A new blood vessel from the choriocapillaris had penetrated Bruch's membrane (between white arrowheads) at the site indicated by a white arrow. A new retinal pigment epithelium (RPE) layer (asterisks) had formed between the Matrigel deposit and photoreceptor outer segments (OS). **B**: The new blood vessel and the choriocapillaris are clearly shown in a confocal image (red). **C**: The DIC image in A superimposed on the confocal image in **B**. The scale bar represents 20 μm.

We identified new blood vessels in many samples collected 45 days after injection of Matrigel in the posterior half of the injected area. A representative one is shown in Figure 5. This blood vessel originated from the choriocapillaris and crossed Bruch's membrane at a position close to the posterior edge of the Matrigel deposit (Figure 5).

## Discussion

**Figure 6:**
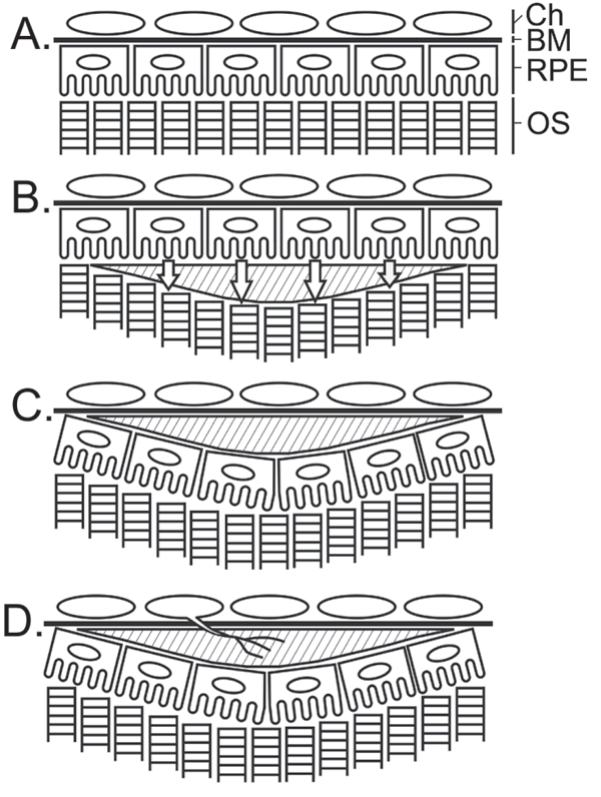
Schematic illustration of RPE translocation and sub-RPE deposit formation. **A**: Normal retina. **B**: A deposit (shade area) has formed in the subretinal space, which disrupts direct contact between photoreceptor outer segment (OS) and the retinal pigment epithelium (RPE). RPE cells leave Bruch's membrane (BM) and migrate toward photoreceptors (indicated by open arrows). **C**: The cells then form a new layer between the deposit and photoreceptors to reestablish RPE-photoreceptor contact, resulting in RPE translocation. The deposit, originally in the subretinal space, becomes a sub-RPE deposit. **D**: Bruch's membrane devoid of RPE attachment is susceptible to invasion of new blood vessels from the choroid (Ch).

The present work has revealed a remarkable ability of RPE cells to reestablish direct contact with photoreceptors when the contact is disrupted by excessive extracellular material in the subretinal space. We have also demonstrated that RPE cells circumvent the subretinal material by translocation and reestablishing a new layer between photoreceptors and the subretinal material, and therefore relocating the subretinal material to sub-RPE space. Our work thus revealed a novel mechanism of sub-RPE deposit formation in which extracellular material first accumulates in the subretinal space, separating the RPE from photoreceptors (Figure 6A,B). To reestablish direct contact with photoreceptors, RPE cells migrate toward photoreceptors and form a new RPE layer, which is equivalent to translocation of the RPE layer. Because of the translocation, there is a switch of positions between the original RPE and the deposit (Figure 6C). Since the RPE is normally used as a reference to describe the surrounding structures, the location of the deposit after RPE translocation is therefore in the sub-RPE space and it becomes a sub-RPE deposit (Figure 6C).

Our work also provides an insight into the mechanism of CNV development. When RPE cells migrate from Bruch's membrane, the RPE-Bruch's membrane complex is dismantled. It seems that the bare Bruch's membrane devoid of RPE is no longer recognized as a barrier and it becomes vulnerable to CNV invasion (Figure 6D). Our data therefore emphasize the importance of RPE as a barrier to CNV invasion, possibly by producing PEDF (pigment epithelium derived factor), a potent antiangiogenic protein [29], and/or through contact inhibition. The observation that cell infiltration is common in samples with CNV (Figure 5A,C) is consistent with pathological findings from AMD patients to suggest an involvement of focal inflammation [24,25]. Furthermore, the deposits may provide a favorable microenvironment for new blood vessels to extend and form a network once they penetrate Bruch's membrane. Therefore, a sub-RPE deposit may serve as a nidus for pathological reactions that lead to CNV development. However, we cannot rule out the possibility that some components in the Matrigel were responsible for inducing CNV to penetrate Bruch's membrane.

Accumulation of extracellular proteins in the subretinal space is commonly seen in patients with exudative retinal detachments, often accompanied by drusen. For example, chronic serous retinal detachment is closely associated with drusen [5]. In addition, serous or exudative retinal detachment in choroidal melanomas is accompanied by drusen-like structures [30-32]. These findings are consistent with the hypothesis that excessive extracellular material in the subretinal space leads to the formation of sub-RPE deposit. Extracellular material accumulation in the subretinal space has also been found in adult vitelliform macular degeneration [33,34]. In our experiments, the subretinal deposit was introduced at a large scale. However, it is conceivable that micro-scaled, clinically undetectable amounts of subretinal deposit could lead to repetitive translocation of the RPE and formation of a macro-scaled sub-RPE deposit.

RPE cells are housekeepers for photoreceptors. They not only are essential for photoreceptor metabolism, but also participate in the RPE-Bruch's membrane complex to form the blood-retinal barrier. It is clear that when the RPE-photoreceptor contact is disrupted, priority is given to reestablishing the RPE-photoreceptor contact rather than maintaining the RPE-Bruch's membrane complex. Our data also provide a clue to how the trophism of RPE cells and the dynamic nature of the RPE-photoreceptor relationship are maintained: a signal from photoreceptors. Although the nature of the signal is yet to be identified, it is highly probable that the messenger is a diffusible factor since it acts across a Matrigel layer. In this regard, it is unlikely that in our experiments Matrigel itself contains a molecule that triggered RPE translocation. In fact, in the absence of photoreceptors, Matrigel failed to stimulate RPE migration. Furthermore, we observed comparable RPE cell translocation using collagen I gel instead of Matrigel (data not shown).

In summary, our present work demonstrates that the presence of a subretinal deposit induces RPE cell translocation, which in turn generates pathological features characteristic of AMD, including formation of the sub-RPE deposit and CNV. These findings indicate a subretinal source of sub-RPE deposits and a key role of RPE translocation in the formation of sub-RPE deposits. Our data also provide evidence that the presence of sub-RPE deposits is sufficient to induced CNV to penetrate Bruch's membrane.
